# Induction of Cell Death Mechanisms and Apoptosis by Nanosecond Pulsed Electric Fields (nsPEFs)

**DOI:** 10.3390/cells2010136

**Published:** 2013-03-06

**Authors:** Stephen J. Beebe, Nova M. Sain, Wei Ren

**Affiliations:** 1 Frank Reidy Research Center for Bioelectrics, Old Dominion University, 4211 Monarch Way, IRP2, Norfolk, Virginia, 23508, USA; E-Mail: novasain@gmail.com; 2 Division of Molecular Carcinogenesis and Targeted Therapy for Cancer, Chinese Academy of Sciences, 1 Beichen West Road, Beijing 100101, China; E-Mail: vivianren@hotmail.com

**Keywords:** apoptosis, caspase-dependent, caspase-independent, Jurkat clones, APAF-1, FADD, N1-S1 hepatocellular carcinoma cells, Ca^2+^ mobilization, mitochondria membrane potential, mitochondria permeability transition pore, cytochrome *c*, electroporation, nanopores 3-10.

## Abstract

Pulse power technology using nanosecond pulsed electric fields (nsPEFs) offers a new stimulus to modulate cell functions or induce cell death for cancer cell ablation. New data and a literature review demonstrate fundamental and basic cellular mechanisms when nsPEFs interact with cellular targets. NsPEFs supra-electroporate cells creating large numbers of nanopores in all cell membranes. While nsPEFs have multiple cellular targets, these studies show that nsPEF-induced dissipation of ΔΨm closely parallels deterioration in cell viability. Increases in intracellular Ca^2+^ alone were not sufficient for cell death; however, cell death depended of the presence of Ca^2+^. When both events occur, cell death ensues. Further, direct evidence supports the hypothesis that pulse rise-fall times or high frequency components of nsPEFs are important for decreasing ΔΨm and cell viability. Evidence indicates in Jurkat cells that cytochrome *c* release from mitochondria is caspase-independent indicating an absence of extrinsic apoptosis and that cell death can be caspase-dependent and –independent. The Ca^2+^ dependence of nsPEF-induced dissipation of ΔΨm suggests that nanoporation of inner mitochondria membranes is less likely and effects on a Ca^2+^-dependent protein(s) or the membrane in which it is embedded are more likely a target for nsPEF-induced cell death. The mitochondria permeability transition pore (mPTP) complex is a likely candidate. Data demonstrate that nsPEFs can bypass cancer mutations that evade apoptosis through mechanisms at either the DISC or the apoptosome.

## 1. Introduction

### 1.1. Therapies for Hallmarks of Cancer

Cancer is a group of diseases that exhibit hundreds of genotypes defined by substantial numbers of mutations and thereby create a major obstacle for treatment. In order to deal with this diversity of cancer diseases, Hanahan and Weinberg [1,2] reasoned that since all mammalian cells express similar mechanisms for proliferation, differentiation and death, most cancers should share a limited number of common hallmarks that govern their behavior. These hallmarks include sustaining proliferation, evading growth suppressors, resisting cell death, enabling replicative immortality, inducing angiogenesis, activating invasion and metastasis, reprogramming energy metabolism and evading immune destruction [2]. These hallmarks occur on a background of genetic instability that can lead to additional mutations. In addition, inflammatory responses by innate immune cells support several hallmarks, thereby promoting malignancies. Identifying these hallmarks provides a context to design targeted cancer therapies that interfere with specific molecules that play critical roles in proliferation and cell death. Over the last several years, significant progress has been made to understand the pathobiology of many cancers and although a number of treatments have been designed based on this knowledge, outcomes so far -have been limited.

### 1.2. Physical Treatments that Target Whole Tumors

Because of treatment resistances and relapses, combining targeted therapy with classical chemotherapy or radiation has become more common and is now a mainstay of cancer treatment [3]. However, there are some emerging therapies that have received limited attention, most likely because they are less conventional treatments. These include several local or regional treatments that target whole tumors by physical approaches including cryotherapy, radiofrequency ablation (RFA), electrochemotherapy (ECT) [4,5], electro-gene therapy (EGT) [6,7], irreversible electroporation (IRE) [8] and nanosecond pulse electric field (nsPEF) ablation [9,10,11,12,13]. RFA provides a relatively effective treatment choice for patients with non-resectable HCC [14], but ablation near major vessels and ducts is contraindicated. ECT is a common treatment in Europe for melanoma cutaneous metastases and clinical trials are on-going for treatment of liver metastases, bone metastases and soft tissue sarcomas [15]. EGT has successfully completed phase I clinical trials for treating melanoma with delivery of IL-12 [16] and evidence of responses in adjacent untreated lesions suggest there is at least a limited systemic response [17]. IRE ablation of locally advanced pancreatic cancer tumors appears to be safe and feasible for unresectable, locally advanced disease [18]. An early analysis of IRE treatment of perivascular malignant hepatic tumors demonstrates safety for treating liver malignancies; however, larger and longer follow-up studies are necessary to determine long-term efficacy [19].

The last four modalities mentioned here - ECT, EGT, IRE and nsPEFs - all use electric fields for treatment. However, they are used very differently for each therapy. ECT is used to electroporate tumor cell plasma membranes in the presence of poorly permeable chemotherapeutic agents such as bleomycin, a glycopeptide antibiotic drug that causes DNA breaks. EGT also uses electroporation, but here it is designed to deliver genes that have anticancer activity or to boost the immune system. It is important that cell plasma membrane “pores” are repaired so cells function to express the delivered gene. IRE extends electroporation by increasing electric fields (to low kV/cm) such that plasma membranes of tumor cells cannot recover from permeabilization and cells die primarily by necrosis. In contrast, nsPEFs extend electroporation by not only increasing the electric field (to tens of kV/cm), but also by decreasing pulse durations from microseconds or milliseconds used in ECT, EGT and IRE, into nanosecond durations. This not only creates pores in plasma membranes, but also in intracellular membranes. Furthermore, unlike electroporation pores, nsPEFs generate large numbers of nanopores in all cell membranes—a phenomenon called supra-electroporation [20,21]. These unique characteristics are proposed to be responsible for apoptosis induction as well as other cell death mechanisms, which have been shown with nsPEFs in several cell types and tumor tissues [9,22,23,24,25,26,27,28]. Initially, intracellular granules were identified as nsPEF targets, but it could only be speculated whether apoptosis was induced as a result of intracellular effects [29]. The presence of apoptosis was demonstrated in studies soon after effects were observed in intracellular structures [9,22]. DNA damage was shown in fibrosarcoma tumors that exhibited tumor growth inhibition using early electrode designs [9]. However, whether DNA damage is a cause or a result of cell death by apoptosis is still unanswered. Others have demonstrated DNA damage [10,12,13,30,31], but it has not been directly linked to a cause of cell death. The latter study demonstrated considerable nuclear membrane and telomere damage as well, suggesting mechanisms other than poration are possible to induce cell death. In this same study and in two others [23,32] changes in cell morphology, phosphatidylserine externalization and caspase activation were demonstrated in cultured cells *in vitro*, but effects on corresponding intracellular structures were not analyzed. The presence of caspase activation and cytochrome *c* release into the cytosol suggested effects on mitochondria, but it was not determined whether this was a direct or indirect effect. Several studies indicated release of intracellular Ca^2+^ [24,32,33,34,35] and evidence for the ER as a possible Ca^2+^ release site [24,33,34]. It was suggested, but not proven, that nsPEFs modulated cell function through intracellular signal transduction mechanisms. This was based on finding that when nsPEF that were well below the threshold for PI uptake and apoptosis, effects were observed that were similar to purinergic agonist-mediated Ca^2+^ release from intracellular stores, which secondarily initiated capacitive Ca^2+^ influx through store-operated Ca^2+^ channels in the PM. It was also suggested that nsPEFs acted as anon-ligand agonist to induce intracellular signaling [24,25,36] based on these observations. While studies above indicated release of cytochrome *c* from mitochondria [22], other studies indicated mitochondrial-independent mechanisms in HCT116 cells that lead to caspase activation and cell death in the presence or absence of p-53 and Bax [25] and without release of cytochrome *c* in the presence of active caspases [26]. Mitochondria were also shown to be a possible intracellular target for cell death as indicated by loss of ΔΨm in several different cell types using several different methods [26,27,37,38]. Again, while some of these show parallel dissipation of ΔΨm and active caspases [26,27], they did not show which event was responsible for the other. 

In the studies here, we used N1-S1 hepatocellular carcinoma (HCC) cells to investigate effects of nsPEFs on subcellular structures and cell viability. We also used Jurkat clones that were deficient in one of three apoptosis-related proteins, FADD, caspase-8 and APAF-1 [39,40,41], to investigate pathways for nsPEF-induced apoptosis.

## 2. Results and Discussion

### 2.1. NsPEFs Induce Nanopores in Plasma Membranes

Early papers published using pulse power with nsPEFs on mammalian cells suggested that effects on intracellular structures occurred without permanent disruption or permeabilization of plasma membranes [29,33]. This was based on a simple electrical model for biological cells, which predicted that because pulse durations were shorter than the plasma membrane charging time, there were increasing probabilities for electric field interactions with cell substructures. When nsPEFs were applied to human eosinophils loaded with calcein, intracellular granules were breached without apparent effects on plasma membranes [29]; that is, without calcein leaking out or propidium iodide (PI) entering through plasma membranes [33]. When Ca^2+^ was imaged in real-time in Jurkat cells exposed to nsPEFs, or ultra-short high-field electric pulses, there were increases in cytosolic Ca^2+^ concentrations within milliseconds [33]. These were the first demonstrations of a broadening of conventional electroporation to include effects on intracellular membranes. This phenomenon was further supported by demonstrating that longer pulses (100 μs and 10 μs durations) resulted in rapid permeability changes with homogeneous magnitudes in surface membranes typical of electroporation. In contrast, shorter pulses (300 ns and 60 ns durations) caused temporally delayed surface membrane permeability changes that were heterogeneous in magnitude [42]. Intracellular effects of nsPEFs were also supported by showing differential permeabilization of lipid vesicles based on differences in charging times of the vesicle membrane capacitance and selective permeabilization of large intracellular vesicles without observably affecting plasma membranes [43]. 

While effects on intracellular structures were easily measured, the apparent absence of plasma membrane effects was due to the creation of pores on the order of nanometers, referred to as nanopores. This was predicted through modeling using a transport lattice approach for electric field effects on cell membranes to induce large numbers of pores in all cell membranes. This effect was designated supra-electroporation [20,21]. The presence of nsPEF-induced nanopores was demonstrated experimentally as voltage-sensitive and inward-rectifying membrane pores [44]. These membrane pores had ion-channel-like properties that were mostly impermeable to propidium iodide. Since nsPEFs affect intracellular membranes, it was supposed that these were also nanopores in nature. Using various cell types loaded with a thallium-sensitive fluorophore, nsPEF-treated cells exhibited increases in intracellular thallium before responding to PI, demonstrating that nanopores were smaller than 1.0–1.5 nm [45]. Van der Waals diameter for thallium is about 0.39 nm and Ca^2+^ is about 0.46 nm [46].

Understanding the formation, size and life-time of lipid membrane pores have been greatly served by computer simulation with Molecular Dynamics (MD) [47,48,49,50]. In many cases, MD simulations have been supported by experimental evidence. In other cases, they have provided models that predict molecular movement of molecules in plasma membranes, which can be analyzed for consistency with other models and with results from experimental analysis of artificial bilayers and living cells. There is general agreement that the primary role in pore formation is played by water dipoles as they create water defects and as they increasingly interact with the electric field at the lipid-water interface; initial steps in pore formation do not depend on the nature of lipid headgroups. The reorientation of the water molecules at the water bilayer interface is relatively fast. The limiting step to the complete reorganization of bilayers is the translocation of lipid head groups inside the hydrophobic lipid domain [47,48,49,50,51,52]. Switching off electric fields appears to allow a complete resealing and reconstitution of bilayer, with the limiting step being the dissociation of the headgroup-headgroup interactions in the membrane core [48]. It has also been shown that there is no pore formation near a membrane channel inserted into the bilayer, which was attributed to a stabilization of the anchoring lipid head groups to the side chains of the channel [48]. Results suggested that there was no distortion of the ion channel formed by hydrogen bonds with the peptide ring [48]. This is expected to be the case for both long duration pulses and nanosecond pulses [53].

MD simulations have been correlated with experimental observations of phosphatidylserine externalization in lipid bilayers [49]. While experimental observations of these events cannot be obtained with nanosecond resolution, MD discerned molecular events of nsPEF-driven pore formation and phosphatidylserine externalization. Nanopores were formed within nanoseconds when electric fields of appropriate magnitudes were applied to cells. While hydrophilic, nanometer-diameter, aqueous pores were forming, phosphatidylserine was pulled electrophoretically from the inner leaflet to the outer leaflet of the lipid membrane.

In another study, MD provided insight into the life cycle of electropores. Pore life-time was divided into discrete stages of pore creation (initiation, construction and maturation) and pore annihilation (destabilization, degradation, deconstruction and dissolution) [50]. The electric field gradient across the membrane determines the pore creation time; the higher the electric field, the faster pores form. Some stages of pore life-time are more electric field-dependent than others. Pore initiation, formation of a water column across the membrane interior, is the major electric field-dependent step in pore creation. Pore construction and maturation as well as pore degradation, deconstruction and dissolution, are not dependent on electric fields. The magnitude of the electric field that initiates pore formation is a weak determinant of pore annihilation, which is much longer lived than pore formation time. Pore destruction time takes about 75 times longer than pore construction. Generally, pores created at higher electric fields are less ordered than those formed at lower electric fields. Pore annihilation may depend on the structure of the pore, which is affected by pore-initiating electric fields. This study was in general agreement with previous stochastic pore hypotheses of electroporation effects [51,52], but further enhanced that model by providing multiple electric field-dependent and -independent stages in pore creation and annihilation. 

**Figure 1:**
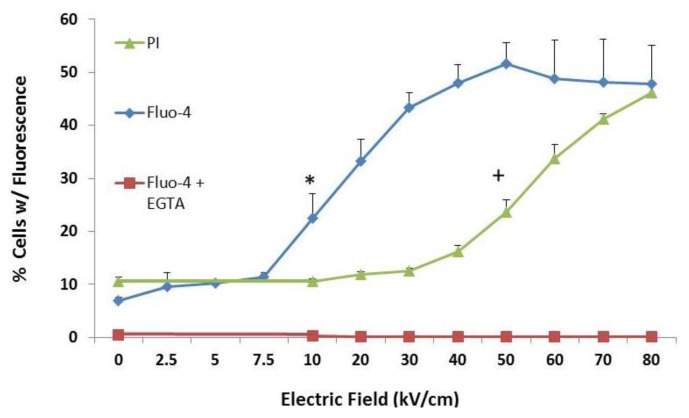
Nanosecond pulsed electric fields (NsPEFs) can induce nanopores larger than Ca^2+^ ions and smaller than PI molecules. N1-S1 HCC cells were treated with one 600 ns pulse with a rise-fall time of 15 ns at the electric fields indicated on the X-axis. Cells were loaded with Fluo-4 Direct as described in the Experimental Section. PI was added immediately after pulse treatments and cells were analyzed by flow cytometry 10 minutes after treatment. The Y-axis indicates the percentage of cells that exhibit fluorescence for either Fluo-4 or PI. When present, EGTA was 5 mM. Statistical significance is indicated at electric fields ≥ those electric fields with symbols; (;* p < 0.001 *vs.* PI control + p < 0.001 *vs.* Fluo-4 control (ANOVA with Tukey correction; n = 3). These data have been previously published in a different format [54].

To examine effects of nsPEFs on plasma membranes and intracellular membranes, cells were loaded with Fluo-4 Direct to determine increases in intracellular Ca^2+^ and treated with single 600 ns pulses with a rapid rise-fall time at various electric fields. PI was added immediately after pulsing (Figure 1). Experiments were also carried out with Fluo-4 in the absence (blue line, diamonds) and presence (red line, squares) of EGTA to chelate all extracellular Ca^2+^. Figure 1 shows that intracellular Ca^2+^ was not detected in the absence of extracellular Ca^2+^, indicating there was no observable release of Ca^2+^ from intracellular stores. Intracellular Ca^2+^ release had been observed in other cells [24,32,33,34,35], so it is not clear if this was typical of N1-S1 cells or if Ca^2+^ release is transient and absent when observations were made or if Fluo-4 is not sensitive enough to detect intracellular Ca^2+^ release. Intracellular Ca2+ is likely present in microenvironments where endoplasmic reticulum (ER) and mitochondria are in close contact. Thus, in these studies, an increase in Fluo-4 fluorescence was used as a marker for small ion, Ca^2+^ transport (0.46 nm) and PI was used as a marker for larger ion transport (1.0–1.5 nm) across plasma membranes. As electric fields were increased between 7.5–10 kV/cm (blue, diamonds), there were significant increases in cells with elevated Fluo-4 fluorescence, indicating small pores in plasma membranes. As electric fields were further increased up to 50 kV/cm, elevated Fluo-4 fluorescence plateaued with about half of cells exhibiting increases in Ca^2+^ as an apparent maximum response under these conditions. PI entry (green line, triangles) into cells was not observable until between 40–50 kV/cm. As electric fields were increased up to 80 kV/cm, numbers of PI positive cells increased in an electric field-dependent manner. This indicates that between 7.5 and 50 kV/cm, nanopores between 0.46 nm and 1.5 nm were present before PI entered cells. Thus, nanopores are formed in response to nsPEFs with pulses as long as 600 ns, but as electric fields increased, so do pore sizes, suggesting that pores can expand with higher electric fields.

### 2.2. Transient Features in nsPEFs Differentially Modulate Intracellular Functions

**Figure 2:**
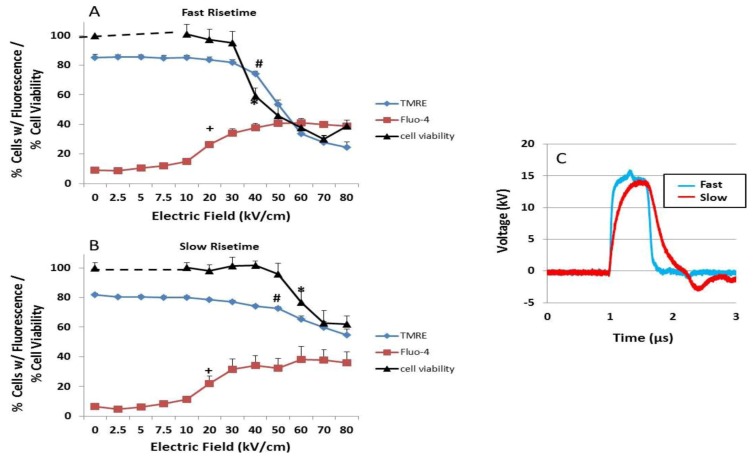
NsPEF fast rise-fall times target ΔΨm and cell viability—(A and B) N1-S1 HCC cells were loaded with Fluo-4 Direct to determine Ca^2+^ influx (red squares) and with TMRE to determine ΔΨm (blue diamonds) by flow cytometry as described in the Experimental Section. The Y-axis indicates the percentage of cells with fluorescence for either Fluo-4 or TMRE, which were determined 10 minutes after nsPEF treatment. In parallel experiments, the percentage of viable cells (black triangles) was determined 24 hours after treatment as described in the Experimental Section. (C) Cells were treated with one 600 ns pulse with a 15 ns fast rise-fall time waveform (blue line, panel A) or a 150 ns slow rise-fall time waveform (red line, panel B) at the indicated electric fields. For Panels A and B significant differences from control sham treatment are indicated for all electric fields ≥ the symbol for Fluo-4 (+p < 0.001, n = 3,); for TMRE (**# **p < 0.03, n = 3) and cell viability (* p < 0.001, n =3 ). (Correction: ANOVA with Tukey’s correction). These data have been previously published in a different format [54].

The hypothesis concerning effects of ultra-short pulses on intracellular membranes is related to the charging time / frequency of the plasma membrane, which is in the range of 70-100 ns or 10-14 MHz in the frequency domain [29]. For pulses longer than the charging time of plasma membranes, such as the 600 ns pulses (1.67 MHz) used in Figure 1, it is hypothesized that a fast *versus* a slow rise-fall time (or transient higher *vs.* lower frequency components) would make a difference as to whether or not intracellular effects are observed. In Figure 2, this hypothesis was directly tested by using single 600 ns pulses (1.67 MHz) with rise-fall times of 15 ns (67 MHz) or 150 ns (6.7 MHz), considerably faster and slower, respectively, than the plasma membrane charging time. To observe changes in plasma membranes, Fluo-4 was used to observe influxes of Ca^2+^ 10 minutes after treatment. As shown in Figure 1, there was no measurable release of Ca^2+^ from intracellular stores. To observe changes in intracellular membranes, TMRE was used to measure the mitochondria membrane potential (ΔΨm) 10 minutes after treatment. Cell viability was determined 24 hours after treatment. 

In both 15 ns (Figure 2A) and 150 ns (Figure 2B) rise-fall time pulses, there were electric field-dependent increases in cells with elevated intracellular Ca^2+^. All electric fields above 20 kV/cm for the 15 ns and 150 ns pulses were statistically significant for increases in intracellular Ca^2+^ (+). As electric fields were increased, there was dissipation in ΔΨm that was statistically significant at electric fields ≥40 kV/cm for the 15 ns rise-fall time pulse (#). For the 150 ns rise-fall time pulses, ΔΨm was dissipated, but not significant until ≥70 kV/cm. For both 15 ns and 150 ns pulses, cell viability decreased parallel to dissipation of ΔΨm. These data support the concept that fast rise-fall time pulses have a greater probability to affect intracellular structures shown here as a decrease in ΔΨm. Furthermore, the intracellular effect of the fast rise-fall time pulse was an apparent determinant of cell viability [54]. 

**Figure 3:**
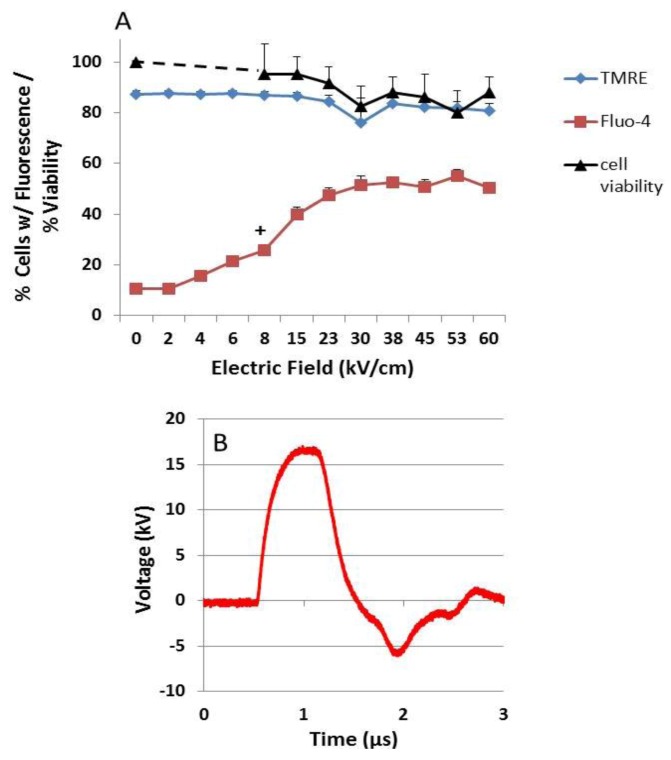
NsPEF effects on ΔΨm determine viability – (A) N1-S1 HCC cells were loaded with Fluo-4 Direct to determine Ca^2+^ influx (red squares) and with TMRE to determine ΔΨm (blue diamonds) as described in the Experimental Section. The Y-axis indicates the percentage of cells with fluorescence for either Fluo-4 or TMRE, which were determined by flow cytometry 10 minutes after nsPEF treatment. In parallel experiments, the percentage of viable cells (black triangles) was determined 24 hours after treatment as described in the Experimental Section. (B) Cells were treated with one 600 ns pulse with a 150 ns slow rise-fall time waveform (red line, panel B) with a mismatched load. Statistical differences *vs.* sham control (0 kV/cm): All n = 3; No significance with cell viability or with TMRE; for Fluo-4, all electric fields ≥ 8 kV/cm, *p < 0.03. ANOVA Tukey’s correction.

By using a slow, 150 ns pulse rise-fall time and introducing a slightly mismatched load, a bipolar tail occurred at the end of the pulse waveform, and a more dramatic difference was observed between plasma membrane and intracellular membrane effects (Figure 3). Using the same experimental design as in Figure 2, Figure 3 shows that Ca^2+^ influx was electric field-dependent and statistically significance between 7.5 and 10 kV/cm, significantly lower than either rise-fall time pulses in Figure 2. In contrast, there were no statistical significant decreases in either ΔΨm or viability at any electric field tested under these conditions. This clearly shows that effects on mitochondria, specifically decreases in ΔΨm, are primarily responsible for cell death; increases in intracellular Ca^2+^ through plasma membranes alone, or release from intracellular stores alone, were not responsible for cell death. Figure 2, Figure 3 also show that transient features resulting from pulse rise-fall times have major effects on intracellular structures / functions, but not on plasma membranes. Furthermore, fast pulse rise-fall times of nsPEFs are primary determinants of intracellular effects. Since there were no decreases in cell viability 24 hours after treatment, the results also indicate that for pores in the range of 1.5 nm generated under these conditions (see Figure 1), cells were able to repair these pores and had sufficient levels of ATP for repair processes at least 24 hours after nsPEF treatment. 

An explanation as to why this pulse waveform has effects on Ca^2+^ influx but not on ΔΨm requires further analysis. As shown in Figure 2, the slow rise-fall time does not have as great an effect on ΔΨm as the fast rise-fall time waveform; rise-fall times (frequency components) appear to be one factor. Another factor that likely contributes to differences between the slow rise-fall time waveforms in Figure 2B, Figure 3 is the mismatched load presumably contributing to a “bipolar tail” at the end of the pulse in Figure 3. This suggests that intricacies of pulse waveforms can have significant impact on cell structures and functions. As others have postulated in a “two hit” hypothesis [55,56], two stimuli are required to open the mPTP including a noxious stimuli such as increases in ROS and elevated Ca^2+^ levels. This ultimately results in mitochondrial Ca^2+^ overload and loss of ΔΨm. Based on this hypothesis and the available data from this study, it is suggested that the pulse itself, an increase in ROS [57], or an effect of the pulse on Ca^2+^ -dependent structure such as the mPTP complex or a component of it, is sufficient to serve as a second hit with elevated Ca^2+^ as the first (or vice versa). It is also known that concentrations of Ca^2+^ required are highly dependent on the prevailing cellular conditions, such as oxidative stress, adenine nucleotide depletion, elevated phosphate concentrations and ΔΨm [58,59]. These results show that there are subtleties about the pulse waveforms that have significant impact on ΔΨm, and perhaps other intracellular structures, but much less effect, if any, on plasma membranes.

### 2.3. NsPEF-Induced Ca^2+^ Mobilization and Cell Viability

**Figure 4:**
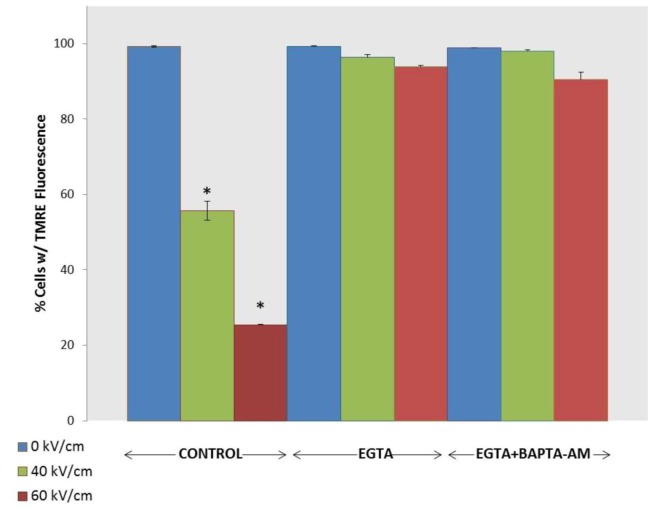
NsPEF-induced dissipation of ΔΨm is Ca^2+^-dependent. N1-S1 cells were loaded with TMRE as indicated in the Experimental Section. Cells were then treated with one 600 ns pulse with a rise-fall time of 15 ns at either 40 or 60 kV/cm or sham treated (0 kV/cm). The Y-axis indicates the percentage of cells that exhibited fluorescence for TMRE as a measure of ΔΨm, which was determined by flow cytometry 10 minutes after treatment in the presence or absence of BAPTA-AM (20 μM) and/or EGTA (5 mM). The symbols indicate treatments that were significantly different (p < 0.05, n = 3) from sham treatment (0 kV/cm). There were no significant difference between treatments with EGTA and EGTA/BAPTA-AM compared to sham treated controls (0 kV/cm).

Calcium as a second messenger can have myriad of effects on cell functions. While studies show that nsPEFs affect cell plasma membranes and intracellular membranes, what functions are regulated by Ca^2+^ from intracellular and/or extracellular sources in response to nsPEFs? NsPEF-induced Ca^2+^ release from the ER exhibited similar kinetics and appeared to target the same inositol 1,4,5-trisphosphate- and thapsigargin-sensitive Ca^2+^ pools in the ER that are activated by the purinergic agonist UTP [24,34]. These receptors secondarily initiate capacitative Ca^2+^ influx through store-operated Ca^2+^ channels in plasma membranes. This led to the concept that nsPEFs could act as a non-ligand agonist and modulate cell functions through intracellular signal transduction mechanisms [24,25,36]. This was exemplified by nsPEF-induced mobilization of Ca^2+^ and modulating neutrophil functions [35], activation of human platelets [36], excitation of cardiac myocytes [60] and activation of Ca^2+^-mediated activation of AMP-activated protein kinase signaling, which responds to cellular energy status [61]. A new Ca^2+^ function is shown by its requirement for nsPEF-induced dissipation of ΔΨm (Figure 4).

Figure 3 showed that even when there were no nsPEF-induced effects on ΔΨm or cell viability, effects on plasma membranes, and possibly on intracellular membranes, led to increases in intracellular Ca^2+^ levels. Several observations led to an interest to determine whether there would be a decrease in ΔΨm if there were no extracellular Ca^2+^. First, nsPEFs induced formation of nanopores in cell membranes that increase intracellular Ca^2+^ levels (see Figure 1). In addition, mitochondria are known to participate in intracellular Ca^2+^ compartmentalization and Ca^2+^ overload of mitochondria can lead to cell death [62,63]. Further, opening the Ca^2+^- and voltage-dependent mitochondria permeability transition (mPTP) causes dissipation of ΔΨm [64]. 

To determine whether nsPEF-induced dissipation of ΔΨm is Ca^2+^-dependent, Figure 4 shows experiments where the ΔΨm was determined using TMRE when N1-S1 HCC cells were preincubated in either EGTA or both EGTA and BAPTA-AM, which chelates extracellular and intracellular Ca^2+^, respectively. Cells were then sham treated or treated with single 600 ns pulses with a rise time of 15 ns at either 40 or 60 kV/cm. The ΔΨm was determined 1, 10 and 30 minutes after treatment. Experiments with BAPTA-AM alone are not shown because it cannot chelate all Ca^2+^ coming from both extracellular and intracellular sources; values at 40 and 60 kV/cm were not significantly different (p < 0.05) than those values in the absence of BAPTA-AM and/or EGTA. Results are shown in Figure 4 for the 10 minute time point. Both 40 kV/cm and 60 kV/cm induced a rapid, electric field-dependent decrease in ΔΨm in significant populations of cells. The ΔΨm continued to decrease by another 10–20 % of cells 30 minutes after treatment. In the presence of EGTA and EGTA & BAPTA-AM, there were no significant decreases in ΔΨm; the absence of Ca^2+^ extinguished the effects of nsPEFs on ΔΨm, indicating that nsPEF-induced decreases in ΔΨm are Ca^2+^-dependent. Similar results were shown for two different Jurkat clones, E6.1 and A3 (data not shown); although some Ca^2+^-independent loss of ΔΨm was observed in Jurkat cells, the loss was significantly Ca^2+^-dependent. These results have significant implications for determining mechanisms for nsPEF effects on mitochondria and for cell viability (Figure 3). Effects on ΔΨm were near immediate, which would be expected if nsPEFs caused nanoporation of inner mitochondria membranes; based on the supra-electroporation hypothesis [20,21]. However, nanoporation of inner mitochondria membranes is not expected to be Ca^2+^-dependent. Thus, nsPEFs appear to have effects distinct from nanopore formation. A possible explanation for these results is that nsPEF-induced dissipation of ΔΨm is due to activation of the mPTP complex, a complex protein structure spanning the inner and outer mitochondria membranes, or the membrane in which it is imbedded. Additional studies are needed to fully address this issue.

### 2.4. Possible Cellular Targets and Cell Death Pathways for nsPEFs

After the initial paper presented evidence that nsPEF affected intracellular structures [29] and considering that cell viability was decreased by nsPEFs [22], other studies were designed to investigate what effects nsPEFs had on cell death mechanisms. Since nsPEFs were shown to be effective in decontamination of bacteria and amelioration of biofouling [65], it was reasoned that nsPEFs could eliminate cancer cells by inducing apoptosis, especially given the impact on intracellular structures and their functions. As indicated in the Introduction, several studies [9,22,23,24,25,26,27,30,31,32,33,34,35,37,38] identified several indicators of apoptosis such as caspase activation as well as effects on cell structures including phosphatidylserine externalization, Ca^2+^ release from intracellular stores, DNA damage and dissipation of ΔΨm in a number of cell types. However, none of these structural / functional effects were directly linked to cell death and it remained to be determined whether cell death was initiated by direct nsPEF effects on these structures or indirect effects as a result of membrane poration and metabolic changes or both direct and indirect effects. 

**Figure 5:**
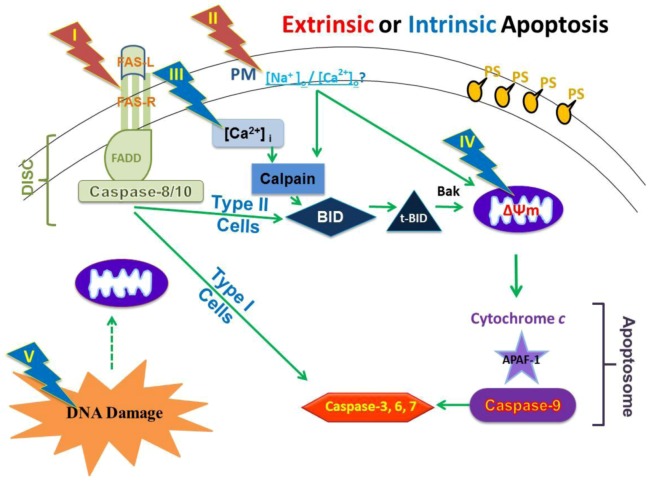
A model for determining nsPEF effects on cellular targets and apoptosis pathways. See text for details.

More directed towards studies that are reported here, a series of *in vitro* studies with HCT116 human colon carcinoma [25], B16f10 mouse melanoma [26] and mouse E4 squamous carcinoma cells [27] demonstrated nsPEFs induce apoptosis, which is often evaded in cancer cells [1,2]. In addition, nsPEFs induced apoptosis equally in HCT116p53(+/+) and HCT116p53(-/-) cells, suggesting that cell death could occur without p53-mediated responses to DNA damage [25]. However, in studies with E4 squamous carcinoma cells [27] and B16f10 cells [26], which did and did not release cytochrome *c*, respectively, elements of extrinsic and intrinsic apoptosis pathways were expected as well as other cell death mechanisms. To systematically investigate specific nsPEF targets and to determine cell death mechanisms, a study was carried out using human Jurkat clones that exhibited deficiencies in apoptosis-related proteins [28]. Although it was previously demonstrated that nsPEF-induced Jurkat cell death was coincident with cytochrome *c* release and caspase activation [22], it was not determined which initiator caspases were activated, whether a type II cell extrinsic and/or an intrinsic apoptosis mechanism was operative, whether cell death was actually dependent on caspase activation, or which cellular sites were targets for nsPEFs. Therefore, consideration was given to whether nsPEFs targeted one of more of five different cellular components for cell death induction (Figure 5). These components were (I) aggregation of the Fas receptor in plasma membranes; (II) permeabilization of plasma membranes, which included effects of sodium and Ca^2+^ influx on other cellular systems; (III) permeabilization of the ER, which would release Ca^2+^ and affect other systems, including calpain activation, stress responses and mitochondria function; (IV) permeabilization of the inner mitochondrial membrane or opening the mitochondria permeability transition pore (mPTP), which would result in dissipation of the ΔΨm; and/or (V) DNA damage, which could affect other systems. Suspected pathways included 1) activation of the death induced signaling complex (DISC) with caspase-8 directly to caspase-3 activation as observed in type I cells, 2) activation of DISC with caspase-8-mediated Bid cleavage and tBid activation of mitochondria responses as observed in type II cells, 3) activation of mitochondria responses for cytochrome *c* release, apoptosome formation with APAF-1, caspase-9 and then caspase-3 activation. This last pathway could be initiated directly at mitochondria or mitochondria response to other internal signals such as protein unfolding response and/or DNA/nucleus damage signals to mitochondria.

### 2.5. NsPEFs Do Not Induce Cell Death Through the Extrinsic Apoptosis Pathway in Jurkat Cells

The first possibility tested whether the extrinsic pathway was activated by nsPEFs. This would cause an aggregation of Fas receptors, which are coupled to the DISC, comprising intracellular Fas receptor domains, FADD and caspase-8. In practice, the question was asked whether nsPEFs required the DISC to induce cell death. Caspase-3/7, -8 and -9 activities were determined in Jurkat clones that were wildtype (Wt) or deficient in caspase-8 (ΔC-8) or deficient in FADD (ΔFADD). Caspase activities were determined to be time-dependent as well as electric field-dependent (data not shown). Caspase-9 and caspase-3 activities were weakest in ΔC-8 clone. Caspase-8 was only weakly activated in the Wt clone and ΔFADD; as expected, caspase-8 was essentially absent in the ΔC-8 clone. 

**Figure 6:**
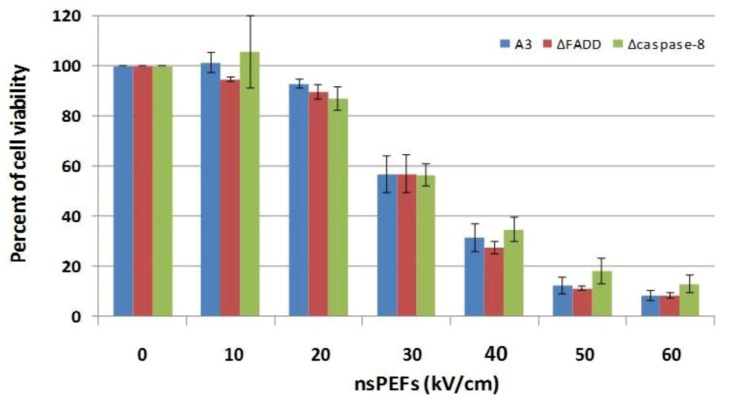
NsPEF-induced cell death in Jurkat clones does not require the DISC. Jurkat clones that are deficient in FADD (ΔFADD) or deficient in caspase-8 (Δcaspase-8) as well as the wildtype clone (A3) were treated with ten 60 ns pulses at 60 kV/cm and assayed for cell viability 24 hours after treatment as described in the Experimental Section. There were no significant differences among clones at any electric field. These data have been previously published in a different format [28].

When cell viability was determined for each clone with increasing electric fields, it was determined that there were no differences in electric field-dependent decreases in cell viability; cell death in all clones exhibited identical electric field dependences (Figure 6). Electric field LD_50_ values were between 30 and 40 kV/cm and pulsing at 60 kV/cm resulted in about 10% survival in all three clones. Thus, cell death in Jurkat clones treated with nsPEFs appeared to be independent of DISC or other mechanisms using FADD or caspase-8. This is in contrast to survival of Fas-stimulated ΔFADD Jurkat cells [66], which we reproduced (data not shown). However, Jurkat cell death occurred when caspases were inhibited by z-VAD-fmk, which sensitizes cells to caspase-independent cell death pathway(s) with other apoptosis stimuli [66,67]. This has been characterized as programmed necrosis [68,69]. This indicates that like other apoptotic stimuli, nsPEFs can activate more than one cell death program, possibly programmed necrosis or necroptosis in the absence of active caspases. 

In order to determine more convincingly whether FADD and/or caspase-8 were involved in nsPEF-induced apoptosis, cytochrome *c* release was investigated in the presence and absence of the pan-caspase inhibitor z-VAD-fmk using Wt, ΔFADD and ΔC-8. If caspase-8 activation, which requires FADD for DISC formation, led to cytochrome *c* release, it should be inhibited by z-VAD-fmk. However, the caspase inhibitor had no effect on cytochrome *c* release with any clone [27]. This argued against the possibility that nsPEFs activated the Fas receptor or that any DISC-mediated mechanism was involved in activating the type II cell pathway in Jurkat cells.

### 2.6. NsPEFs Induce Intrinsic Caspase-Dependent at Lower Electric Fields (20-40 kV/cm) and Caspase-Independent Cell Death at Higher Electric Fields (≥ 50 kV/cm) in Jurkat Cells

Overall, these data indicate that nsPEF-induced cell death signals do not appear to pass within the typical type I pathway through DISC/caspase-8 to caspase-3 nor travel through the typical type II pathway through DISC/caspase-8/ tBid to cytochrome *c*. This suggested that signals pass within the apoptosome pathway. To answer this question, a Jurkat clone was used that was deficient in APAF-1 by silencing with a siRNA plasmid permanently transfected into Jurkat clone E6.1 [39,40,41]. 

To confirm that APAF-1 was not functional, cells were stimulated with ten 60 ns pulses at 60 kV/cm and assayed for caspase-9 and caspase-3 catalytic activity in both vector control and the APAF-1 deficient clones. The vector control exhibited robust caspase-9 and caspase-3 catalytic activity that peaked 6 hours after treatment and the APAF-1 deficient cells had no activity for either caspase (data not shown). This confirmed the work of Shawgo *et al.* [39]. In the vector control, nsPEFs activated caspase-3/7 in an electric field-dependent, biphasic manner. There was no caspase-3 activation at 10 kV/cm, although there were electric field-dependent increases in activity up to 40 kV/cm. However, at higher electric fields, caspase-3 activity was no greater then control at 60 kV/cm [28].

**Figure 7:**
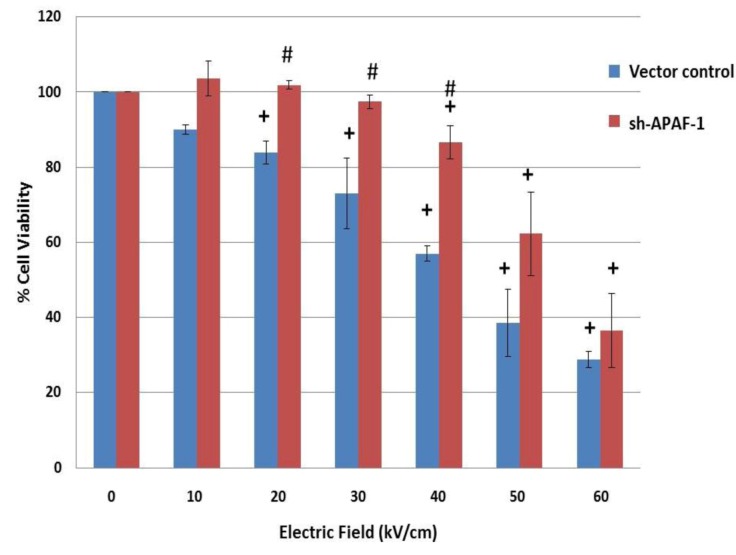
NsPEF-induced cell death is caspase-dependent at lower electric fields (20–40 kV/cm) and caspase-independent at higher electric fields (≥ 50 kV/cm) at in Jurkat cells: A Jurkat clone deficient in APAF-1 and its vector control were tested with ten 60 ns pulses with increasing electric fields. Cell viability was determined 24 hours later - as described in the Experimental Section. The same results were obtained with the MTS assay. For statistical significance: + p < 0.05 *vs.* sham control (0 kV/cm); # p< 0.05 vector control clone *vs.* sh-APAF-1 clone; (n = 3). These data have been previously published in a different format [28].

To determine cell death under these same conditions, cells were analyzed for survival 24 hours after treatment with nsPEFs (Figure 7). Cell death in the vector control was electric field-dependent with a threshold for cell death at 20 kV/cm, a LD_50_ between 40 and 50kV/cm and 70% cell death at 60 kV/cm. In contrast, cell death in ΔAPAF was shifted significantly to higher electric fields; the threshold for cell death was between 40 and 50 kV/cm with an electric field LD_50_ between 50–60 kV/cm. This indicates that at these electric fields, nsPEF-induced cell death is APAF-1- and caspase-3-dependent and suggests that cell death is induced at least in part by apoptosis at electric fields between 10 and 40 kV/cm. However, at 60 kV/cm when caspase-3 activity was similar to control [28], cell death was not significantly different between vector control and ΔAPAF. This indicates that at electric fields ≥ 50 kV/cm, cell death is caspase-independent. 

### 2.7. NsPEFs Activate Bid but Do Not Alter Levels of Other Endogenous Bcl-2 Family Proteins

**Figure 8:**
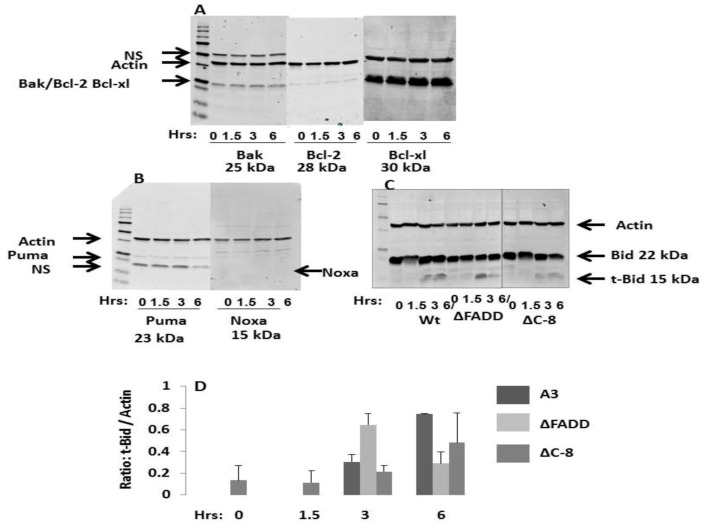
NsPEFs induce Bid cleavage in a time-dependent manner in Jurkat cells. Cells were sham treated or exposed to ten 60 ns pulse at 60 kV/cm. Samples were prepared for immunoblot analysis at indicated times after pulsing. Extracts (50 μg protein) were separated by SDS-PAGE, transferred to PVDF membranes and probed with antibodies against various antigens (in kDa) ***A****)*. Bak (25), Bcl-2 (28), Bcl-xl (30) and Actin (45) in the Wt clone; ***B****).* Puma (23), Noxa (15) and Actin (45) in the Wt clone; ***C****).* Bid (22), which also recognized t-Bid (15), and Actin (35) in three indicated clones. ***D****). *Bid and t-Bid from Jurkats A3 wildtype, FADD deficient (ΔFADD) and caspase-8 deficient (ΔC-8) were quantified using Odyssey infrared imager, normalized to total Bid levels in control and expressed as the ratio of fluorescence of t-Bid to fluorescence of Actin, a loading control. Values, mean ± SE (n=3) from experiments like that shown in ***C****.*

Some reports have indicated that nsPEFs increase or decrease the levels of endogenous Bcl-2 family proteins [25,27,70]. To determine whether nsPEFs affected pro- or anti-apoptotic proteins in human Jurkat clones, immunoblots were analyzed when cells were treated with ten 60 ns pulses at 60 kV/cm using antibodies against pro-apoptotic Bak (Bax is not expressed in Jurkat cells), anti-apoptotic Bcl-2 and Bcl-xl (Figure 8A) and anti-apoptotic BH3 only proteins Puma and Noxa (Figure 8B). In addition, since Bid is downstream of FADD and caspase-8, Wt, ΔFADD and ΔC-8 clones were analyzed for Bid cleavage (Figure 8C, D). The only protein level that changed in response to this lethal nsPEF condition was t-Bid. Within the first 6 hours after treatment, Bak levels did not change nor did Bcl-2 levels, which was expressed at relatively low levels. Bcl-xl, which was highly expressed, did not change nor did levels of Puma, which were expressed at low levels. Noxa levels were not detected. The results with Puma and Noxa, which are expressed in response to direct DNA damage [71], suggest that DNA damage may not be a major response to nsPEFs in Jurkat cells. Figure 8C shows a typical time course immunoblot for Bid and t-Bid and Figure 8D shows results from a series of immunoblot experiments. T-Bid was not present in sham-treated control cells, but increased in a time-dependent manner when cells were exposed to a lethal nsPEF condition. Interestingly, ΔFADD exhibited the greatest increase in Bid cleavage reaching a peak around 3 hours and decreasing by 6 hours, while the Wt continued to increase through the 6 hour time course. ΔC-8 clone exhibited the weakest activation of t-Bid, but was nevertheless present. 

### 2.8. Bid Cleavage Is Sensitive to Inhibition of Caspases and Calpains in E4 Squamous Carcinoma and Jurkat Clones

In nsPEF-induced cell death in E4 squamous cell carcinoma cells, Bid cleavage is only partially inhibited by EGTA indicating a Ca^2+^-dependent component as well as a Ca^2+^-independent component [27]. Unlike Jurkat cells, cytochrome *c* release in E4 cells was caspase-dependent (~50%–60%) as well as caspase-independent (~50%). This clearly demonstrated at least two different mechanisms for cytochrome *c* release. In E4 cells, it was hypothesized that the caspase-independent mechanism was due to calpain activity, which has been demonstrated [72,73]. Using the calpain substrate Ac-LLY-AFC, it was shown that Ca^2+^-dependent and some Ca^2+^-independent calpain activity was indeed present [27]. Calpain could play a role in apoptosis through cleavage of Bid to t-Bid, which would then lead to cytochrome *c* release. Since caspase activity was shown to be Ca^2+^-independent [26], the Ca^2+^-dependency of Bid cleavage, assumed to be due to calpain activity, was tested in the absence of caspase activity [27] These studies indicated that 60–70% of the Ca^2+^ contributing to Bid cleavage comes from extracellular sources through plasma membranes and 30%–40% comes from intracellular stores.

**Figure 9:**
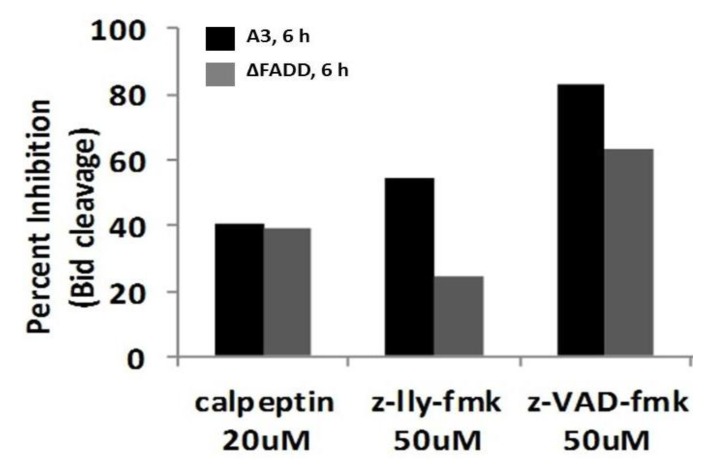
NsPEFs induce caspase- and calpain-dependent Bid cleavage in Jurkat cells. Jurkat A3 wildtype (dark bars) and a Jurkat clone deficient in FADD (ΔFADD) (light bars) were pre-incubated for 30 min in the presence of calpeptin (20 μM), z-LLY-fmk (50 μM), and z-VAD-fmk (50 μM) prior to nsPEF treatment with ten 60 ns pulses at 60 kV/cm or sham treated. Cell lysates were prepared 3 hours post pulse, separated by SDS-PAGE like experiments shown in Figure 8C and Bid and t-Bid were quantified using Odyssey infrared imager and normalized to total Bid levels in control. Results were plotted as the percent inhibition of Bid cleavage in the absence of inhibitors. Values represent a single experiment.

Since Bid cleavage, which is downstream of FADD and caspase-8, occurred in all clones, it was of interest to determine what was responsible for t-Bid formation in the absence of DISC. Since Bid is known to be activated not only by caspases, but also by calpains [27,72,73], it was determined whether activation of Bid was sensitive to blockade by protease inhibitors. Figure 9 shows an experiment that analyzed formation of t-Bid by immunoblot analysis. Because ΔC-8 exhibited lowest levels of t-Bid, quantification was not determined. When calpains were inhibited by calpeptin and z-LLY-fmk 40%–60% and 25%–40% inhibition was observed in Wt (dark bars) and ΔFADD (light bars), respectively, suggesting that calpains were partially responsible for Bid cleavage. Caspase inhibition by the pan caspase inhibitor z-VAD-fmk inhibited Bid cleavage by 60%–80%. These results indicate that nsPEFs activate Bid by cleavage with both calpains and caspases. In other experiments, the calpain inhibitors calpeptin and PD150606 had no effect on cytochrome *c* release (data not shown). Therefore, unlike results from E4 cells [27], t-Bid induced by nsPEFs in Jurkat cells does not appear to be effective for cytochrome c release in response to activation by either caspases or calpains. Caspase cleavage of Bid must be downstream of cytochrome *c*, presumably by caspase-3 after activation by caspase-9.

### 2.9. Hypothesized Cell Targets and Cell Death Pathways Activated by nsPEFs

**Figure 10:**
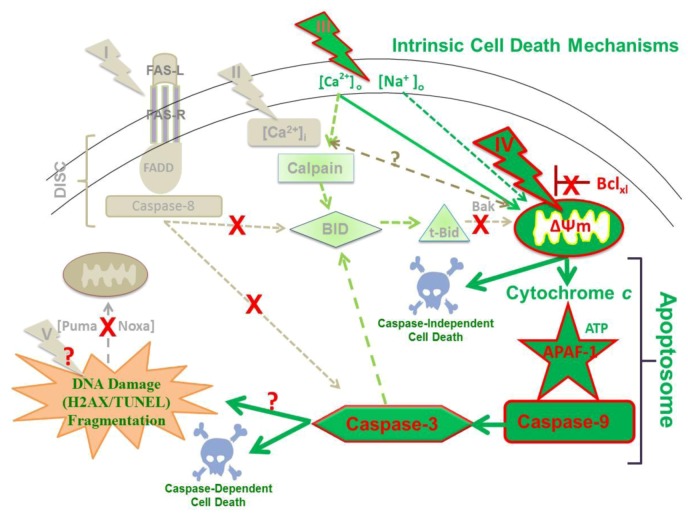
A model for nsPEF-induced cell death in human Jurkat cells.

Figure 10 gives an overview of our present understanding of cell death mechanisms induced by nsPEFs, at least in Jurkat cells. The dark green elements and arrows show primary effects and the light green elements and arrows show other events that are involved as cells die in response to nsPEFs. The mitochondria are primary targets (Figure 3); effects on ΔΨm are due to time- and Ca^2+^-dependent effects possibly on the mPTP or other Ca^2+^ -dependent protein. However, additional studies are necessary to confirm this. For example, can overexpression of Bcl-2 or Bcl-xl or inhibition of mPTP prevent nsPEF-induced dissipation of ΔΨm? NsPEFs could also open mitochondria apoptosis-inducing channels (MACs) [74] and/or voltage-dependent anion channels (VDAC) [75]. They could also induce mitochondria Ca^2+^ overload [76] or promote death-induced bystander effects of Bak (or Bax) [77]. 

The influx of Ca^2+^ most likely also leads to activation of calpain, which cleaves Bid (Figure 9), yet this does not seem to play a role in cytochrome *c* release because cytochrome *c* release is not affected by calpain inhibitors [28]. Thus, nsPEFs cause a decrease in ΔΨm, especially with fast rise-fall times (Figure 2), a release of cytochrome *c *within 1.5–3 hours after treatment [22,28] and an activation of caspase-9 and -3 [28]. This occurs through formation of the apoptosome, clearly indicating activation of a well-characterized, mitochondria-, APAF-1-mediated apoptosis pathway. In addition, at high electric fields, nsPEF induced caspase-independent cell death (Figure 7). In these studies, caspases appear to cleave Bid (Figure 8C), but this does not lead to cytochrome *c* release [28]. Although DNA damage by double strand breaks was present in this Jurkat cell model as determined by Histone 2AX phosphorylation (data not shown), its role in cell death has not yet been clearly determined. NsPEF-induced DNA damage has been demonstrated in a number of cell types by a number of assays *in vitro* [9,30,31,70] and *in vivo* [12,13]. In the Jurkat model here, the presence of DNA double strand breaks did not induce increases in Puma or Noxa (Figure 8B), suggesting this was not a major mechanism to activate intrinsic apoptosis in response to nsPEF-induced DNA damage. While such damage could lead to cell death, cell death has not been shown to be DNA damage- dependent like cell death has been shown to be dependent on dissipation of ΔΨm or on caspases shown here. Aggregation or other possible mechanisms for activating Fas receptors do not appear to play a role in nsPEF-induced cell death (Figure 6); cytochrome *c* release was not affected by inhibition of caspases with z-VAD-fmk [28], which would be required if cell death signals were initiated through formation of the DISC. Effects of nsPEFs on intracellular Ca^2+^ stores or on influx of Ca^2+^ alone were not responsible for cell death, since Ca^2+^ influx without effects on ΔΨm did not lead to significant cell death (Figure 3). Effects of nsPEFs on the plasma membrane were evident by the influx of Ca^2+^. The presence of nanopores in the plasma membrane was observed when influx of Ca^2+^ could be seen at lower electric fields than influx of propidium iodide (Figure 1).

One of the frequently asked questions about using nsPEFs for cancer treatments is a concern for specificity towards cancer cells while sparing normal, non-cancerous cells. While some cells are more vulnerable to nsPEF cytotoxicity than other cells [30,78,79,80], it remains to be seen if such differences are clinically relevant. A recent report suggesting this evaluated paired tumor and normal cell lines obtained from ATCC from the same individual, using pulses with durations of 30 ns and electric field strengths at 30 kV/cm and repetition rates of 50 Hz [80]. The study indicated that there were tendencies for these pulses to be more cytotoxic to the human basal cell carcinoma (BCC, TE 354.T) cell line compared to its normal sister cell line (TE 353.SK). The BCC cells were less able to recover and exhibited more active caspases than normal sister TE cells. The authors suggest that preferential killing of tumor cells may be less injurious to normal skin cells surrounding BCC tumors. While these results are interesting, there still remains the question of how cancer cells compare to normal cells that are not in continuous culture. Future clinical trials treating BCC and other skin cancers will provide answers to this cancer *vs.* normal specificity for nsPEF cytotoxicity. Studies with nsPEF-treated ectopic B16f10 melanoma tumors showed some transient skin damage, which recovered without scarring [10]. This suggests that nsPEF treatment of skin lesions in patients will heal without scarring. An unpublished clinical trial on human skin provides additional data for the absence of scarring as well as skin discoloration in response to nsPEF treatments.

## 3. Experimental Section

### 3.1. Cell Culture

Wild type Jurkat T-lymphocytes (clone A3) and mutant cell lines deficient for FADD (clone I 2.1) or caspase-8 (clone I 9.2) were purchased from ATCC (Manassas, VA). The clone with APAF-1 silenced (ΔAPAF-1) and its vector control (in Jurkat clone E6.1) were generous gifts from Dr. John Robertson, Department of Pharmacology, Toxicology and Therapeutics, University of Kansas Medical Center, Kansas City, Kansas. These clones were grown and cultured as previously described [39,40,41]. N1-S1 HCC cells were purchased from ATCC and cultured as previous described [54].

### 3.2. Treatment of Cells with nsPEFs

Cells were treated in cuvettes with nsPEFs as previously described [22,24,25,26,27,28]. Jurkat cells were exposed with or without ten pulses with durations of 60 ns (rise-fall times ~5 ns) and electric field strengths ranging from 0 to 60 kV/cm using pulse generators as previously described [22,24,25,26,27,28]. N1-S1 cells were exposed to single 600 ns pulses with electric fields up to 80 kV/cm. Pulse rise-fall times were 15 ns or 150 ns with waveforms [54].

### 3.3. Determination of Propidium Iodide (PI) Uptake

Cells were exposed to nsPEFs, PI was added to a final concentration of 2.5 μg/mL immediately after pulsing and cell are analyzed by flow cytometry 10 minutes after nsPEF treatment.

### 3.4. Flow Cytometry Analysis of Ca^2+^ and Δψm

The levels of intracellular Ca^2+^ were determined using Fluo-4 Direct (Molecular Probes, Eugene, Oregon), which includes a proprietary formulation with probenecid to prevent transport of the fluorophore from the cell. Depolarization of the ΔΨm was detected using tetramethylrhodamine ethyl ester (TMRE) (Immunochemistry Technologies LLC, Bloomington, MN) as previously described [27]. Cells were preincubated with Fluo-4 Direct for 60 min at 37 °C. During the last 15 min of Fluo-4 Direct incubation, 200 nM TMRE was added and incubated for 15 min. Cells were washed, resuspended in culture media, exposed to nsPEFs either a fast (15 ns) or slow (150 ns) rise-fall time and electric fields ranging from 0–80 kV/cm. Flow cytometric analysis was performed 1, 10 or 30 minutes after treatment with nsPEFs (ten minutes times are shown). Analysis by flow cytometry was with Becton Dickinson FacsAria flow cytometer. To determine Δψm in the presence and absence of Ca^2+^, cells were preincubated with or without 20 μM BAPTA-AM and/or 5 mM EGTA for 30 minutes followed by nsPEF treatment. 

### 3.5. Determination of Cell Viability

Cell viability was determined 24 hours after treatment with nsPEFs according to the manufacture’s protocol in the CellTiter-Glo Luminescent Cell Viability Assay Kit (Promega, Madison, WI). The luminescent signals for ATP levels were directly proportional to cell numbers. Luminescence was analyzed in luminometer (Gemini XPS, Molecular Devices, CA). Identical results were determined with the MTS assay. 

### 3.6. Flow Cytometry Analysis of Cytochrome c Release

Cytochrome *c* was assessed using the Innocyte Flow Cytometric Cytochrome *c* Release assay (Calbiochem) as previously described [27]. 

### 3.7. Determination of Bcl-2 Family Proteins by Immunoblot Analysis

At time points after nsPEF treatments, sham (control) and nsPEFs treated cells were collected and washed with cold PBS once and lysed in RIPA buffer (50mM Tris-HCL, 150mM NaCl, 1% NP-40, 0.5% sodium deoxycholate, 0.1% SDS) (Boston BioProducts) for 30 min at 4°C. The lysate were centrifuged at 14,000 rpm for 20 min at 4 °C and the protein content in supernatants was measured using BCA assay kit (Pierce). Equal amounts of protein (50 μg) were separated by SDS-polyacrylamide gel electrophoresis (Any KD™, Bio-Rad), transferred to PVDF membranes and blotted with primary antibodies to Bid/t-Bid (Cell signaling Technology, MA), Bcl-2 (mouse polyclonal, Santa Cruz Biotechnology), Bcl-xl (rabbit monoclonal, Cell Signaling Technology), Bak (rabbit polyclonal, Cell Signaling), Puma (rabbit polyclonal, Cell Signaling) and Noxa (rabbit polyclonal, Abcam) followed by IRDye 680-conjugated secondary antibody (LI-COR Biosciences) in blocking buffer for 1hour. Protein–antibody complexes were quantified on an Odyssey Infrared Imaging System (LI-COR Biosciences). β-Actin (1:1000; Cell signaling Technology) was used as a loading control. When included, the pan caspase inhibitor z-VAD-fmk (50 μM), the calpain inhibitors z-LLY-fmk (50 μM) or calpeptin (20 μM) were pre-incubated for 20 minutes before treatment. 

## 4. Conclusions

NsPEFs affect multiple cell targets including plasma membranes, membranes of intracellular Ca^2+^ stores such as ER, mitochondria and DNA. Based on studies presented here and under these conditions, Ca^2+^-dependent dissipation of Δψm appears to most closely correlate with loss of cell viability. When intracellular Ca^2+^ was elevated without dissipation of Δψm, cells remained viable for at least 24 hours. When Ca^2+^ was absent, there was no significant dissipation of Δψm in N1-S1 HCC or Jurkat clones A3 and E6.1. However, based on the observation that permeabilization effects on plasma membranes for Ca^2+^ influx occur at lower electric fields (~10 kV/cm) than dissipation of Δψm (~40 kV/cm), Ca^2+^ will always be present when nsPEFs dissipate Δψm. The finding that dissipation of Δψm is Ca^2+^-dependent suggests that effects are likely to be on Ca^2+^-dependent protein(s) such as the mPTP complexes than on nanoporation of the inner mitochondria membrane; however, this has not been shown directly. Nevertheless, such a result suggests that nsPEFs can have effects on protein structures as well as on cell membranes. This presents new paradigms for analyzing effects of nsPEFs on cell structures and raises new questions about how electric fields interact with lipids and amino acids to affect cell functions.

The most likely effect of nsPEF-induced Ca^2+^-dependent dissipation of Δψm is caspase-dependent cell death, which occurs at lower electric fields as well as caspase-independent cell death at higher electric fields. The most likely paradigm is dissipation of ΔΨm and cytochrome *c* release causes formation of the apoptosome for activation of caspases -9 and -3, which induces caspase-dependent cell death in some cells while dissipation of Δψm induces caspase-independent cell death in other cells. Because cell death can be caspase-independent, nsPEFs can bypass oncogenic mechanisms the evade apoptosis by blocking formation of the DISC or the apoptosome. This gives explanations for the efficacy of nsPEF for skin cancers [10,11,12,70,81,82,83] and other cancers [9,13,83] *in vivo*.
